# Inflammation Disturbed the Tryptophan Catabolites in Hippocampus of Post-operative Fatigue Syndrome Rats *via* Indoleamine 2,3-Dioxygenas Enzyme and the Improvement Effect of Ginsenoside Rb1

**DOI:** 10.3389/fnins.2021.652817

**Published:** 2021-08-26

**Authors:** Shu Liu, Yue Cheng, Wei-Zhe Chen, Jin-Xiao Lv, Bei-Shi Zheng, Dong-Dong Huang, Xu-Fen Xia, Zhen Yu

**Affiliations:** ^1^Department of Biomedical Sciences, College of Veterinary Medicine and Life Sciences, City University of Hong Kong, Kowloon Tong, Hong Kong, SAR China; ^2^Department of Clinical Laboratory, Tongde Hospital of Zhejiang Province, Hangzhou, China; ^3^Department of Gastrointestinal Surgery, First Affiliated Hospital of Wenzhou Medical University, Wenzhou, China; ^4^Department of Surgery, Shanghai Tenth People’s Hospital Affiliated to Tongji University, Shanghai, China

**Keywords:** post-operative fatigue syndrome, inflammatory cytokine, p38MAPK, NF-κB/p65, IDO, ginsenoside Rb1

## Abstract

**Aim:**

Post-operative fatigue syndrome (POFS) is a common complication that prolongs the recovery to normal function and activity after surgery. The aim of the present study was to explore the mechanism of central fatigue in POFS and the anti-fatigue effect of ginsenoside Rb1.

**Method:**

We investigated the association between inflammation, indoleamine 2,3-dioxygenase (IDO) enzyme, and tryptophan metabolism in the hippocampus of POFS rats. A POFS rat model was induced by major small intestinal resection. Rats with major small intestinal resection were administered ginsenoside Rb1 (15 mg/kg) once a day from 3 days before surgery to the day of sacrifice, or with saline as corresponding controls. Fatigue was assessed with the open field test (OFT) and sucrose preference test (SPT). ELISA, RT-PCR, Western blot, immunofluorescence, and high-performance liquid chromatography (HPLC) were used to test the inflammatory cytokines; p38MAPK, NF-κB/p65, and IDO enzyme expressions; and the concentrations of tryptophan, kynurenine, and serotonin, respectively.

**Result:**

Our results showed that POFS was associated with increased expressions of inflammatory cytokines and p38MAPK and higher concentrations of kynurenine and tryptophan on post-operative days 1 and 3; a lower serotonin level on post-operative day 1; and an enhanced translocation of NF-κB/p65 and the IDO enzyme on post-operative days 1, 3, and 5. Ginsenoside Rb1 had an improvement effect on these.

**Conclusion:**

Inflammatory cytokines induced by large abdominal surgery disturb tryptophan metabolism to cause POFS through the activation of the p38MAPK–NF-κB/p65–IDO pathway in the hippocampus. Ginsenoside Rb1 had an anti-fatigue effect on POFS by reducing inflammation and IDO enzyme.

## Introduction

Post-operative fatigue syndrome (POFS) is a common complication after surgery, especially in major abdominal and cardiac procedures (Rubin et al., 2004). POFS is an unpleasant symptom and has a major impact on a patient’s quality of life, which can be divided into peripheral and central fatigue. The former manifests itself as the decline of skeletal muscle motor function; the latter mainly shows up as mental disorder, such as feelings of frustration, depression, or hopelessness and having difficulty concentrating or being attentive (Christensen et al., 1982; Schroeder and Hill, 1991; Zargar-Shoshtari et al., 2009). For affected patients, the recovery to normal function and activity is prolonged, which contributes to substantial burden to both the patients and their families (DeCherney et al., 2002). Unfortunately, the etiology of the syndrome has not been fully explained.

Conventional studies on central fatigue of post-exercise fatigue have supported the “serotonin hypothesis,” in which tryptophan uptake into the brain enhances serotonin production, thereby resulting in a sleepy feeling after excessive exercise (Schwarcz and Pellicciari, 2002; Yamamoto et al., 2012). Similarly, elevation of the blood tryptophan level was also found in patients and animals with POFS (Lu et al., 2016; Morris et al., 2016). However, since tryptophan taken up into the brain has two metabolic pathways, the serotonin and kynurenine pathways, which account for 5 and 95% of the tryptophan metabolism, respectively, kynurenine possibly plays a vital role in the mechanism of central fatigue (Yamashita and Yamamoto, 2014). Furthermore, in contrast with post-exercise fatigue, systemic inflammation is very common in patients with POFS, especially those who had large abdominal surgery. It has been widely accepted that the enzyme indoleamine 2,3-dioxygenase (IDO) can be activated by inflammatory cytokines and then upregulate the kynurenine pathway, which is responsible for inflammation-induced depression, due to neurotoxicity *via* the production of various active metabolites, such as quinolinic acid (Guillemin et al., 2005; Huang et al., 2020). Therefore, whether large abdominal surgery will induce central nervous system (CNS) inflammation and shift the balance of tryptophan metabolism, and which inflammation pathway mediates this imbalance, need further exploration.

Ginseng, the dried root of *Panax ginseng* C. A. Meyer (Araliaceae), has been used as a tonic to treat various disorders in Chinese traditional medicine. Ginsenoside Rb1 (GRb1), one of the ginsenosides, has been reported to have an anti-fatigue effect (Barton et al., 2010; Lee et al., 2016). In our previous studies, we successfully established a POFS rat model by major intestinal resection and explored the improvement effects of GRb1 on peripheral fatigue by reducing skeletal muscle oxidative stress through the activation of the PI3K/Akt/Nrf2 pathway (Zhang et al., 2011; Zhuang et al., 2014). However, the pharmacological mechanism of GRb1 on central fatigue remains unknown.

In the present study, we explored the pathway between inflammatory cytokines, inflammatory pathways, IDO enzyme, and tryptophan metabolism in the hippocampus of rats undergoing major abdominal surgery and investigated the mechanisms of central fatigue in POFS rats and the improvement effects of GRb1.

## Materials and Methods

### Animals

Adult male Sprague–Dawley rats weighing 225–300 g were purchased from the Shanghai SLAC Laboratory Animal Co., Ltd., Shanghai, China. The rats were maintained in a facility with specific pathogen-free and controlled temperature (20–22°C), humidity (45–55%), and light (12-h light/12-h dark cycle) conditions, with standard rat chow and water made available *ad libitum*, except for 1 day of fasting before and after the operation. The experimental procedures were approved by the Institutional Animal Committee of Wenzhou Medical University. All rats were well-cared for throughout the experiment in accordance with the Guide for the Care and Use of Laboratory Animals.

### Drugs and Chemicals

Ginsenoside Rb1 (GRb1) (purity > 98%) was purchased from Shanghai Tauto Biotech Co., Ltd. (Shanghai, China). All reagents and chemicals were procured from local suppliers and were of analytical grade.

### Experimental Design

After an adaptation period of 1 week, rats were randomly divided into three groups—control group (CG), POFS model group (PG), and GRb1-treated POFS model group (GG)—into four cohorts: post-operative days 1, 3, 5, and 7.

**FIGURE 1:**
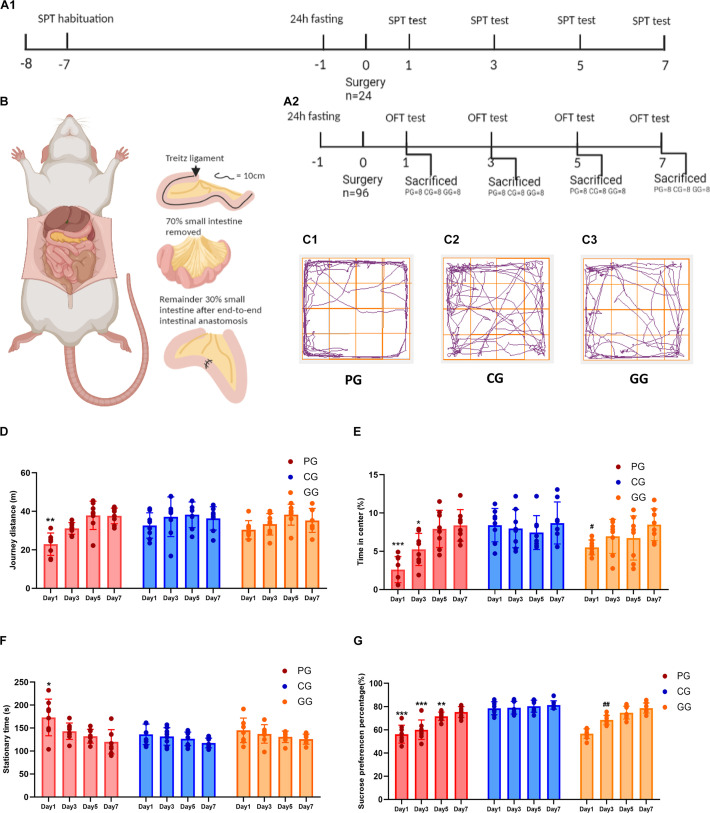
Large abdominal surgery induces fatigue syndromes in post-operative early-stage, which be reversed by GRb1. **(A)** The schema of behavioral experiments process timeline, **(A1)** timeline of sucrose preference test (SPT) (*n* = 8, each group), rats received habituation at -7 day and testing at day 1, 3, 5 and 7 after surgery; fasting 24 h before surgery, 12 h food and water deprivation before every test. **(A2)** Timeline of Open-field test (OPT) (*n* = 96), 96 rats entered into cohort of OPT, fasting 24 h before surgery, and 24 rats (*PG* = 8, *CG* = 8, and *GG* = 8) be sacrificed after OPT in post-operative day 1, 3, 5 and 7, respectively. **(B)** The cartoon schema of 70% intestine resection surgery in PG and GG rats. **(C1–C3)** Representative photographs of rats’ moving trace captured by Any-maze in post-operative day 1 for PG, CG, and GG rats, respectively. **(D)** Journey distance (m) of rats (*n* = 8, each group) in OPT during 5 min. **(E)** The percentage of time (%) rats (*n* = 8, each group) stayed in the central arena in OPT during 5 min. **(F)** The stationary time (s) of rats (*n* = 8, each group) in OPT during 5 min. **(G)** The percentage of sucrose preference (%) of rats (*n* = 8, each group) in SPT. PG, POFS model group; CG, Control group; GG, Ginsenoside Rb1-treated POFS model group; ***p < 0.001, **p < 0.01, *p < 0.05 vs. the control group and *^##^p* < 0.01, *^#^p* < 0.05 vs. the PG at the same time point, respectively (two-way ANOVA with LSD test). Each bar represents mean ± SD.

The POFS rat model was induced by major small intestinal resection as described in a previous study (Zhang et al., 2011). After opening the abdomen, the length of the small intestine was measured. We removed 70% of the small intestine starting from 10 cm below the ligament of Treitz and ending at the terminal ileum. End-to-end intestinal anastomosis can then be conducted while the mesentery is repaired (Figure 1B). Before closing the abdomen, the anastomotic leak was detected by squeezing the intestinal contents. Rats in PG and GG received surgery as mentioned above, while rats in the CG went through the same procedure, without any small intestinal resection. All rats received 24-h fasting but water free before surgery.

Rats in the CG and PG were intraperitoneally administered saline at a dose of 4 ml/kg for 3 days before surgery and once daily after surgery until sacrifice. Rats in GG were administered GRb1 dissolved in saline at a dose of 15 mg/kg (4 ml/kg) using the same method as in the other two groups.

Four cohorts of rats including those in the PG, CG, and GG received the open field test (OFT) on days 1, 3, 5, and 7 after surgery, respectively. Once the OFT finished, the rats were sacrificed and samples of hippocampal tissues were prepared after left ventricular perfusion by saline (Figure 1A2). A special cohort of rats was used for the sucrose preference test (SPT). Rats were deprived of water for 12 h water on the day of surgery, and food and water were suspended for 12 h before every subsequent SPT day (post-operative days 3, 5, and 7) (Figure 1A1).

### Open Field Test

The open field arena was a square black plastic box (100 × 100 × 50 cm^3^) and used an automated video tracking system (ANY-maze, Shanghai, China). We divided the bottom into 16 grids and defined 4 central grids as the center arena. Each rat was placed individually at the corner of the arena and its behavior monitored for 5 min. The journey (the number of grids the rats walked through), stationary time, and time in the center area of each rat were recorded.

### Sucrose Preference Test

After food and water deprivation for 12 h, each rat was exposed to the sucrose solution (1% *w*/*v*) and pure water for 12 h. The positions of the two bottles were exchanged every 6 h to prevent place preference. Fluid consumption was measured by weighing the bottles before and after the test. The sucrose preference percentage (SPP) was calculated using the following formula: SPP (%) = sucrose solution consumption (g)/(water consumption + sucrose solution consumption) × 100%. The same process was performed 7 days before surgery as a habituation.

### Enzyme-Linked Immunosorbent Assay

After the OFT, blood was collected from the postcava and centrifuged at 5,000 rpm for 15 min after allowing to stand for 4 h. Then, the supernatant was collected and placed in a refrigerator at −80°C until subsequent testing. According to the manufacturer’s instructions, the concentrations of interleukin-1β (IL-1β), IL-6, and tumor necrosis factor alpha (TNF-α) in the serum were determined by ELISA kits (PI303, PI328, and PT516; Beyotime, Shanghai, China).

### Real-Time Reverse Transcription Polymerase Chain Reaction

Real-time quantitative polymerase chain reaction (PCR) assays were performed to quantitate the expressions of the inflammatory cytokines IL-6, IL-1β, and TNF-α (*n* = 8 for the CG, PG, and GG). The β*-actin* gene, which is a housekeeping gene, was chosen as the internal gene control. Total RNA was extracted from the isolated hippocampus of rats using the TRIzol reagent (Invitrogen, Carlsbad, CA, United States). RNA purity and concentration were assessed with UV spectrophotometry (2.0 > *A*_260_/*A*_280_ > 1.8). Complementary DNA (cDNA) was synthesized using RT kit (Toyobo, Osaka, Japan). The PCR reaction mixture was prepared using the SYBR Green Real-time PCR Master Mix-Plus (Toyobo, Osaka, Japan). The primer sequences were designed by the Shanghai Sangon Biological Engineering Technology and Services Co., Ltd. (Shanghai, China). Amplification of cDNA was performed with a 7500 Real-Time Quantitative PCR instrument (Applied Biosystems, Carlsbad, CA, United States). The reaction parameters were incubated at 95°C for 1 min, then 40 cycles at 95°C for 15 s, 60°C for 15 s, and 72°C for 1 min. All reactions were performed in triplicate and normalized using β-actin as an endogenous control gene. Relative data quantitation was performed by the 2^–ΔΔCT^ method.

### Western Blot

Western blotting was performed on proteins from hippocampal tissue homogenates (*n* = 8 for the CG, PG, and GG). The hippocampal cytoplasm and nuclear protein were extracted using a nuclear extraction kit (Pierce, Rockford, IL, United States) according to the manufacturer’s instructions. Total protein was also extracted from the hippocampus using a protein extraction kit (Beyotime, Nanjing, China). The protein concentrations were determined using a Micro BCA Protein Assay kit (Beyotime, Nanjing, China). Thirty micrograms of protein per specimen was separated on a sodium dodecyl sulfate polyacrylamide gel electrophoresis (SDS-PAGE) and blotted onto a polyvinylidene difluoride (PVDF) membrane (Millipore, Billerica, MA, United States). The membranes were blocked for 1.5 h with Tris-buffered saline with Tween (TBST) containing 5% non-fat dry milk at room temperature and incubated overnight with the primary antibodies at 4°C, washed, and then incubated with the appropriate secondary antibodies [horseradish peroxidase (HRP)-conjugated anti-IgG antibody, 1:5,000; Biosharp, Hefei, China] for 1 h at room temperature. After washing, the protein bands were visualized using enhanced chemiluminescence (ECL) and exposed to X-ray film. After scanning the X-ray film, the pixel density was calculated with Quantity One software ver. 4.6.2 (Bio-Rad, Hercules, CA, United States). The primary antibodies were: anti-IDO (1:200, sc-53978; Santa Cruz Biotechnology, Dallas, TX, United States), anti-p38MAPK (1:1,000, 8960s; Cell Signaling, Danvers, MA, United States), anti-phospho-p38 (p-p38) (1:250, 4511s; Cell Signaling), anti-NF-kappaB/p65 (NF-κB/p65) (1:1,000, 8242s; Cell Signaling), anti NF-kappaB/phosphor-p65 (NF-κB/pp65) (1:250, 3033s; Cell Signaling), and anti-GAPDH (1:1,000; Hangzhou Goodhere Biological Technology Co., Ltd., Hangzhou, China), anti-β-actin (1:1,000; Hangzhou Goodhere Biological Technology Co., Ltd.), or anti-lamin-B1 (1:500; BioWORLD, Dublin, OH, United States) polyclonal antibody used as a loading control.

### Immunofluorescence

The hippocampus was dissected out, imbedded in Tissue-Tek (Sakura, Torrance, CA, United States), and frozen in liquid nitrogen (*n* = 8 for the CG, PG, and GG). Sections of 10 μm thick were cut using a frozen section machine. After fixation in paraformaldehyde (4%), the sections were incubated with the primary antibodies overnight, rabbit anti-NF-κB/p65 (1:100, 8242s; Cell Signaling, Danvers, MA, United States), followed by incubation with the secondary antibody Dylight 488 anti-rabbit (1:500; Abcam, Cambridge, United Kingdom). Immunoreactivity was visualized at the appropriate wavelengths with a light and an epifluorescence microscope (Nikon 80i, Tokyo, Japan).

### High-Performance Liquid Chromatography

Samples of hippocampal tissue were weighed and dissected before homogenizing at 4°C with 0.2 M perchloric acid including 100 μm EDTA-2Na in a Teflon/glass homogenizer (*n* = 8 for the CG, PG, and GG). The homogenate was centrifuged at 4°C for 15 min at 20,000 × *g*. The supernatant was collected and filtered through a 0.45-μm centrifuge tube filter before injection into the high-performance liquid chromatography (HPLC) system. Determination of the concentrations of tryptophan, kynurenine, and serotonin in hippocampal tissue homogenates was performed using a Hypersil C18 column (250 mm × 4.6 mm i.d., 5 mm particle size; Elite Analytical Instrument Co., Ltd., Dalian, China). The concentrations of kynurenine and tryptophan were determined according to the method described by Wang and Tang (2006) and Herve et al. (1996). Briefly, the mobile phase for fluorescent light detection consisted of 220 mmol/L zinc acetate/acetic acid solution containing 5% (*v*/*v*) acetonitrile, pH 6.0. The flow rate was set at 1.5 ml/min. The injected sample volume was 20 μl. The excitation wavelength was 365 nm and the emission wavelength was 480 nm for kynurenine; for tryptophan, these were 254 and 404 nm, respectively. Determination of serotonin concentration was according to the method recorded by Szeitz and Bandiera (2018). Briefly, the mobile phase consisted of 100 mmol/L sodium acetate/acetic acid solution containing 10% (*v*/*v*) methanol, pH 5.0. The flow rate was set at 1.0 ml/min. The injected sample volume was 20 μl. The excitation and emission wavelengths were 330 and 250 nm, respectively.

### Statistical Analysis

All values were expressed as the mean ± standard deviation (SD). Statistical analysis was performed using two-way ANOVA, followed by *post-hoc* tests (using the least significant difference test, LSD-t) for multigroup comparisons. A value of *p* < 0.05 was considered statistically significant.

## Results

### Large Abdominal Surgery Induces Fatigue Syndromes in Post-operative Early Stage, Which Can Be Reversed by GRb1

We used the OFT and SPT to judge whether large abdominal surgery will induce fatigue syndrome and depression-like behaviors and whether GRb1 will reverse them. The results of the OFT showed that, in the early stage after surgery (post-operative day 1), rats in the PG had fewer journeys and longer stationary times on the first day after surgery during the 5-min test (Figures 1D,F). In addition, rats in the PG showed significantly less exposure in the center of the open field arena on post-operative days 1 and 3, which could be improved by GRb1 (Figures 1C,E).

The SPT was used to assess the rats for loss of pleasure. From the results, we found that the SPP was significantly decreased after the large abdominal surgery procedure in the PG rats. Compared with those in the CG, rats in the PG showed obviously lower levels of SPP on post-operative days 1–5, and in the first 3 days after surgery; SPP decreased to below 65%, which reached the criterion for anhedonia. After intervention with GRb1, rats in the GG recovered markedly on post-operative day 3 (Figure 1G).

### Inflammatory Cytokines Are Upregulated in POFS Rats and Relieved by GRb1 in Serum and Hippocampus

**FIGURE 2:**
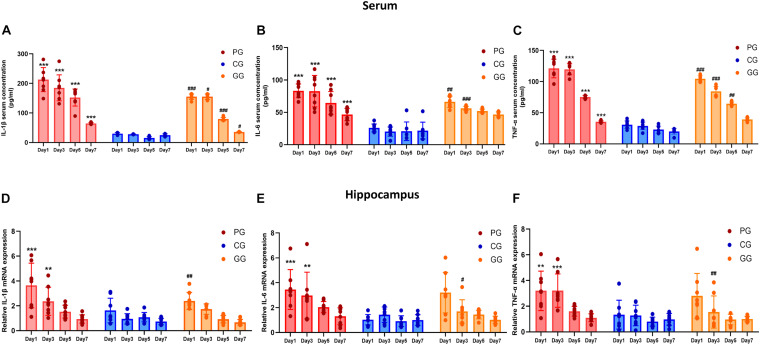
Inflammatory cytokines are upregulated in POFS rats and relived by GRb1 in serum and hippocampus. **(A–C)** ELISA detects the Level of IL-1β, IL-6, and TNF-α (pg/ml) in rats’ serum (*n* = 8, each group), respectively. **(D–F)** RT-PCR detects the mRNA expression fold change of IL-1β, IL-6, and TNF-α in rats’ hippocampus (*n* = 8, each group), respectively. PG, POFS model group; CG, Control group; GG, Ginsenoside Rb1-treated POFS model group; ***p < 0.001, **p < 0.01 vs. the control group and *^###^p* < 0.001, *^##^p* < 0.01, *^#^p* < 0.05 vs. the PG at the same time point, respectively (two-way ANOVA with LSD test). Each bar represents mean ± SD.

To investigate post-operative inflammation in both peripheral blood and the CNS and to explore the link between them, we performed ELISA and real-time PCR to assess the levels of inflammatory cytokines in serum and the hippocampus, respectively. Serum cytokine concentrations showed obvious and lasting increases in rats in the PG; conversely, GRb1 decreased the concentrations of IL-1β, IL-6, and TNF-α. Compared with rats in the CG, the serum IL-1β, IL-6, and TNF-α levels in the PG were significantly increased from day 1 to day 7 after surgery, while rats in the GG showed obvious attenuation than those in the PG at different post-operative time points (Figures 2A–C).

The expressions of inflammatory cytokines in the hippocampus revealed the same increasing trend as those in serum, but only showed significant difference in the early period after surgery. On post-operative days 1 and 3, the expressions of IL-6, IL-1β, and TNF-α messenger RNA (mRNA) in the PG rats were significantly upregulated compared with those of rats in the CG. In addition, GRb1 downregulated the mRNA expressions of IL-1β on post-operative day 1 and of IL-6 and TNF-α on post-operative day 3 (Figures 2D–F). These results suggest that 70% of major small intestinal resection will result in systemic inflammation. Dramatic and long-lasting increases of peripheral blood cytokines may act as the link between surgery and inflammation in the hippocampus. In addition, hippocampal inflammation was serious on post-operative days 1 and 3, then gradually recovered to the CG level; GRb1 played an alleviating role in the first 3 days after surgery.

### p38MAPK-NF-κB/p65 Pathway Activated in POFS Rats’ Hippocampus and Attenuated by GRb1

**FIGURE 3:**
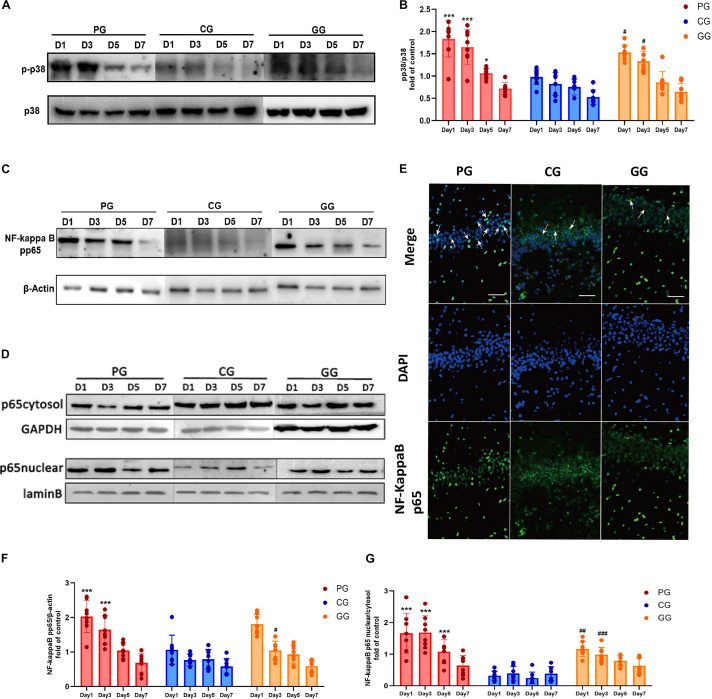
p38MAPK-NF-κB/p65 pathway activated in POFS rats’ hippocampus and be attenuated by GRb1. **(A)** Representative bands of western blot of pp38MAPK and p38MAPK in different groups. **(B)** Levels of the relative expression of pp38MAPK/p38MAPK in hippocampus (*n* = 8, each group). **(C)** Representative bands of western blot of NF-κB/pp65 and β-actin in different groups. **(D)** Representative bands of western blot of NF-κB/p65 in cytoplasm and GAPDH in different groups; Representative bands of western blot of NF-κB/p65 in nucleus and lamin B in different groups. **(E)** Immunofluorescence detects the NF-κB/p65 transposition to nucleus in hippocampus on post-operative day 1. NF-κB/p65 (green), nucleus (DAPI, blue), scale bar, 50 μm, white arrows stand for NF-κB/p65 and DAPI merged. **(F)** Levels of the relative expression of NF-κB/p65/β-actin in hippocampus (*n* = 8, each group). **(G)** Levels of the relative expression of NF-κB/p65 in nucleus/NF-κB/p65 in cytoplasm in hippocampus (*n* = 8, each group). PG, POFS model group; CG, Control group; GG, Ginsenoside Rb1-treated POFS model group; ***p < 0.001, *p < 0.05 vs. the control group and *^###^p* < 0.001, *^##^p* < 0.01, *^#^p* < 0.05 vs. the PG at the same time point, respectively (two-way ANOVA with LSD test). Each bar represents mean ± SD.

To further explore the mechanism of activation of the inflammation pathway in the hippocampus of POFS rats, we examined the protein expressions of phospho-p38MAPK and NF-κB/phospho-p65. In addition, NF-κB/p65 nuclear translocation was detected by both immunofluorescence and nuclear protein extraction followed by Western blot. Rats in the PG, which had large abdominal operation, showed significantly increased phospho-p38MAPK expression than did rats in the CG on post-operative days 1, 3, and 5. Compared with rats in the PG, those in the GG obviously downregulated the phospho-p38MAPK protein expression on days 1 and 3 after surgery (Figures 3A,B).

The expression of NF-κB/phospho-p65 had a similar change to phospho-p38MAPK. Compared with rats in the CG, those in the PG revealed significantly upregulated expressions on post-operative days 1 and 3, while the GG rats showed a reversion effect on day 5 after surgery (Figures 3C,F). The ratio of NF-κB/p65 protein expression in the nucleus and cytoplasm illustrates the activation of the NF-κB/p65 pathway from another direction. The PG rats showed significantly higher ratios than did the CG rats on post-operative days 1, 3, and 5, while GRb1 obviously inhibited the nuclear translocation of NF-κB/p65 on post-operative days 1 and 3 (Figures 3D,G). In immunofluorescence, an obvious upregulation of the translocation of NF-κB/p65 from the cytoplasm into the nucleus in the PG has been detected, accompanied with a modest upregulation in the GG and less translocation in the GG (Figure 3E). These results revealed that large abdominal surgery and sequenced neuroinflammation will result in p38MAPK and NF-κB/p65 phosphorylation and promote NF-κB/p65 to translocate from the cytoplasm into the nucleus.

### IDO Mediated the Tryptophan Degradation Pathway Activated in POFS Rats’ Hippocampus and Reversed by GRb1

**FIGURE 4:**
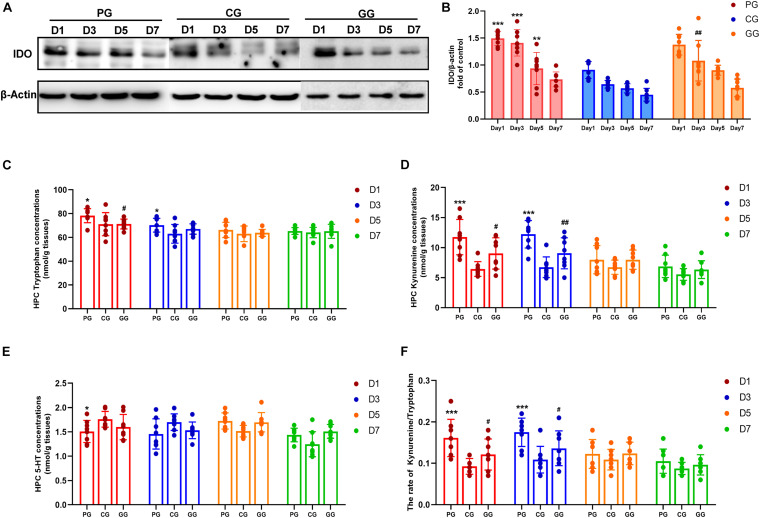
Indoleamine 2,3-dioxygenase (IDO) mediated Tryptophan degrading pathway activated in POFS rats’ hippocampus and be reversed by GRb1. **(A)** Representative bands of western blot of IDO and β-actin in different groups. **(B)** Levels of the relative expression of IDO/β-actin in hippocampus (*n* = 8, each group). **(C)** HPLC detects the concentration of tryptophan in hippocampus (nmol/g tissue) (*n* = 8, each group). **(D)** HPLC detects the concentration of kynuramine in hippocampus (nmol/g tissue) (*n* = 8, each group). **(E)** HPLC detects the concentration of serotonin (5-HT) in hippocampus (nmol/g tissue) (*n* = 8, each group). **(F)** The ratio of kynuramine/tryptophan (%) in hippocampus (*n* = 8, each group). PG, POFS model group; CG, Control group; GG, Ginsenoside Rb1-treated POFS model group; ***p < 0.001, **p < 0.01, *p < 0.05 vs. the control group and*^ ##^p* < 0.01, *^#^p* < 0.05 vs. the PG at the same time point, respectively (two-way ANOVA with LSD test). Each bar represents mean ± SD.

Based on the effect of the inflammatory response on tryptophan metabolism, we examined the expression of the key enzyme IDO in the tryptophan pathway and its metabolism productions: kynurenine and serotonin. The protein expression of IDO was detected by Western blot in the hippocampus. The results showed that large abdominal operation increased the expression of IDO significantly on days 1–5 after surgery. Meanwhile, GRb1 obviously downregulated IDO expression on post-operative day 3 (Figures 4A,B).

Indoleamine 2,3-dioxygenase (IDO) is the rate-limiting enzyme in the tryptophan–kynurenine metabolic pathway. HPLC was used to detect the levels of tryptophan, kynurenine, and serotonin. Firstly, tryptophan concentration in the hippocampus of the PG rats was shown to be significantly increased on post-operative days 1 and 3, while rats in the GG showed a decreasing trend on the first day after surgery (Figure 4C). Secondly, the level of kynurenine was enhanced significantly in the PG compared to the CG on days 1 and 3 after surgery and was attenuated at the same time points in rats in the GG (Figure 4D). Serotonin in the hippocampus showed a relatively stable level and only had an obvious increase on post-operative day 1 in the PG rats (Figure 4E). Furthermore, although tryptophan, the metabolism material in the IDO-mediated pathway, had been upregulated in the early stage after surgery, the ratio of kynurenine/tryptophan still showed a dramatic increase on post-operative days 1 and 3; the ratio in the GG rats was significantly lower than that in the PG rats at these time points (Figure 4F).

## Discussion

In the present study, we have provided evidence that a marked peripheral inflammation induced by large abdominal surgery will cause inflammatory cytokines to increase in the hippocampus of rats, which upregulated the kynurenine pathway in tryptophan metabolism mediated by the overexpression of the IDO enzyme through the p38MAPK–NF-κB/p65 pathway. The shift of tryptophan balance results in fatigue behaviors in post-operative rats, especially at the early stage after surgery. Ginsenoside Rb1 protected the brain from neuroinflammatory damage by inhibiting the expression of the inflammatory cytokines.

Post-operative fatigue syndrome (POFS) is a common complication following major abdominal surgery. It is an unpleasant and distressing symptom characterized by muscular weakness, decreased ability to concentrate, increased need for sleep, and, often, some degree of depression (Rubin and Hotopf, 2002; Rubin et al., 2004; Zargar-Shoshtari and Hill, 2009). It is hard to isolate the depressive symptoms from central fatigue, especially chronic fatigue syndromes (CFS). It has been estimated that at least 60% and possibly 70% of those with CFS will also have significant psychiatric conditions, specifically depressive disorders (Larkin and Martin, 2017). In our study, we used the OFT and SPT to assess rats’ fatigue and depressive-like behaviors. At the early stage after surgery, rats who had 70% of the intestine removed lost the exploratory interest in the new environment and pleasure for the sucrose water award, which is in accord with rodents’ depressive-like behavior. On the other hand, except for the depressive-like behavior, the stationary time and the journey in OFT were also decreased, which may be explained by the peripheral fatigue in POFS, especially the muscular consumption.

Several authors have implicated increased levels of inflammatory cytokines in the generation of fatigue in ill individuals (Manjaly et al., 2019; Lanser et al., 2020; Schreiner et al., 2021). Peripheral inflammation is closely associated with CNS inflammation. Inflammatory cytokines in the systemic circulation are actively transported to the brain by transporters in endothelial cells or *via* diffusion through relatively permeable areas of the blood–brain barrier (Goehler et al., 2007; Kobrzycka et al., 2019; Xiong et al., 2019). Meanwhile, signals relating to systemic immune-inflammatory responses also reach the nuclei of the solitary tract through afferent vagal signals induced by the presence of pro-inflammatory cytokines in the gut and the spleen (Xiong et al., 2019), which affect neuron’s recruitment in the hippocampus *via* the neural circuit projection (Suarez et al., 2018). In addition, the hippocampus is part of the limbic system and develops nerve fiber connectivity with emotion-related brain regions, like the cortex and amygdala (Liu et al., 2017). Therefore, it plays a crucial role in mental disorders such as depression and anxiety. Therefore, we chose the hippocampus as the target brain structure to explore central fatigue in POFS. In our study, the dramatic increase of cytokines in serum may be the evidence of link between hippocampal inflammation and surgery.

NF-κB is a protein complex that controls the transcription of DNA (Kim et al., 2006). Inflammatory cytokines can induce overexpression of the IDO enzyme *via* signal transducer and activator of transcription protein (STAT)-independent pathways involving p38MAPK and NF-κB (Fujigaki et al., 2006; Huang et al., 2020; Zhang et al., 2020). Our experiments showed a similar tendency of cytokines, pp38, NF-κB/pp65, and IDO to have upregulated expressions and increased nucleus/cytoplasm ratios on post-operative days 1 and 3, then gradually recovered to normal levels as in the CG, which illustrated the activity of the p38MAPK-NF-κB/p65 pathway promoting the overexpression of the IDO enzyme.

The increase of the tryptophan level in plasma is characteristic of POFS not only in patients but also proven in rodents (Lu et al., 2016; Morris et al., 2016). The mechanism responsible for this is the increase of free fatty acid in plasma after surgery, which prefers to combine with albumin rather than tryptophan. The altered equilibrium between the bound and unbound tryptophan will increase the level of free tryptophan in both plasma and the brain (Yamamoto et al., 1997). This confirmed the tryptophan increase in the hippocampus on days 1 and 3 after surgery. However, different from exercise fatigue, in which the serotonin level is enhanced in the CNS and induces sleepy and dim moods owing to the higher level of tryptophan (Fernstrom and Fernstrom, 2006), fatigue caused by inflammation will promote IDO expression and then shift the tryptophan metabolism to the kynurenine pathway markedly. This process will induce oxidative stress neurotoxic effect through quinolinic acid, the product of kynurenine (Myint, 2012). Our results confirmed this with evidence that the kynurenine level and the ratio of kynurenine/tryptophan in the hippocampus were obviously higher after surgery. The special tryptophan metabolism characteristic during systemic inflammation may explain why, with observations in cancer patients treated with inflammation and fatigue-inducing chemotherapy, fatigue is usually unresponsive to selective serotonin reuptake inhibitors and also brings us a new clinical idea to improve fatigue in POFS patients (Ahles et al., 2002; Bower et al., 2002; Bjorklund et al., 2019).

Ginseng is one of the most commonly used herbal medicines in the world, considered as the king of herbs. GRb1, one of the ginsenosides, has multiple pharmacological activities such as anti-fatigue, anti-oxidation, neuroprotection, anti-inflammation, and anti-diabetes (Du et al., 2011; Shergis et al., 2013). In our experiments, GRb1 had protective effects on the anti-inflammation of both the CNS and peripheral blood. Meanwhile, GRb1 showed its potential in POFS treatment by adjusting the balance of tryptophan–kynurenine metabolism.

## Conclusion

In conclusion, the present study demonstrated that large abdominal operation can elevate the inflammatory cytokines in the hippocampus of rats due to systemic inflammation, enhancing the expression of the IDO enzyme through the activation of the p38MAPK and NF-κB pathways. The increased IDO enzyme interrupted the balance of tryptophan–kynurenine metabolism, which can be the special pathogenesis of POFS. GRb1, one of the ginsenosides in ginseng, has an anti-fatigue effect in POFS induced by major small intestinal resection in rats, which may be related to the suppression of inflammation in the CNS.

## Data Availability Statement

The raw data supporting the conclusions of this article will be made available by the authors, without undue reservation.

## Ethics Statement

The animal study was reviewed and approved by the Wenzhou Medical University animal ethics committee.

## Author Contributions

All authors listed have made a substantial, direct and intellectual contribution to the work, and approved it for publication.

## Conflict of Interest

The authors declare that the research was conducted in the absence of any commercial or financial relationships that could be construed as a potential conflict of interest.

## Publisher’s Note

All claims expressed in this article are solely those of the authors and do not necessarily represent those of their affiliated organizations, or those of the publisher, the editors and the reviewers. Any product that may be evaluated in this article, or claim that may be made by its manufacturer, is not guaranteed or endorsed by the publisher.
